# Retrospective Evaluation of the Impact of the COVID-19 Pandemic on the Incidence of Alopecia Areata in a Single Dermatological Department

**DOI:** 10.3390/jcm15103682

**Published:** 2026-05-11

**Authors:** Łukasz Chętko, Julia Hofmann, Karolina Brzychcy, Marta Matych, Dorota Sobolewska-Sztychny, Marcin Noweta, Bartosz Zakrzewski, Małgorzata Dominiak, Joanna Narbutt, Aleksandra Lesiak

**Affiliations:** 1 Student Scientific Research Club of Experimental, Clinical and Procedural Dermatology, Medical University of Lodz, Pomorska Street 251, 92-213 Lodz, Poland; julia.kolodziejska@stud.umed.lodz.pl (J.H.); karolina.brzychcy@stud.umed.lodz.pl (K.B.); 2 Department of Dermatology, Pediatric Dermatology and Dermatological Oncology, Medical University of Lodz, Pomorska Street 251, 92-213 Lodz, Poland; marta.matych@umed.lodz.pl (M.M.); dorota.sobolewska-sztychny@umed.lodz.pl (D.S.-S.); marcin.noweta@umed.lodz.pl (M.N.); malgorzata.dominiak@umed.lodz.pl (M.D.); joanna.narbutt@umed.lodz.pl (J.N.); lesiak_ola@interia.pl (A.L.); 3 Laboratory of Autoinflammatory, Genetic and Rare Skin Disorders, Medical University of Lodz, Pomorska Street 251, 92-213 Lodz, Poland; 4 Zakrzewscy Clinic of Aesthetic Medicine and Dermatology, ul. Porcelanowa 23b, 40-246 Katowice, Poland; bartosz.zakrz@gmail.com

**Keywords:** alopecia areata, COVID-19, SARS-CoV-2, autoimmune diseases, epidemiology

## Abstract

**Background:** Alopecia areata (AA) is an autoimmune disease characterized by diverse patterns of non-scarring hair loss. Due to its susceptibility to immune dysregulation and psychological stress, there is growing speculation regarding the potential role of SARS-CoV-2 infection and the COVID-19 pandemic in its development, recurrence, or exacerbation. This retrospective study aimed to evaluate patients affected by AA from a single dermatological center, specifically focusing on the impact of the COVID-19 pandemic on hospitalization rates. **Methods:** Data comprising demographic characteristics, disease subtype, number, and duration of hospitalizations were digitized and statistically analyzed. The five-year period prior to the pandemic (2015–2019) was compared with the subsequent four years (2020–2023) to assess any changes. **Results:** The study involved 428 individuals (256 children and 172 adults), with a slight predominance of women (68.2%). The median ages in adults and children were 39.13 years and 8.66 years, respectively. Following the pandemic, there was a 13.81% decrease in the mean age among adult males. Hospitalizations surged by 207.62% after the pandemic, increasing from 223 to 686 admissions. Additionally, the diagnosis of alopecia areata totalis increased significantly by 55.6%. The residential distribution of pediatric patients also shifted notably, with 72.16% residing in urban areas and 27.84% in rural areas between 2020 and 2023. **Conclusions:** The significant increase in hospitalization rates and the diversity of disease subtypes observed in this study may suggest a potential correlation between COVID-19 and the development or altered course of alopecia areata. A deeper understanding of this association could enhance treatment outcomes in dermatology, ultimately improving patient care.

## 1. Introduction

Alopecia areata (AA) is a prevalent autoimmune disorder manifesting as non-scarring hair loss, affecting approximately 2% of the global population [1,2]. The clinical presentation of AA is highly variable, ranging from small, well-defined patches of hair loss to complete loss of hair on the scalp and body. The condition is indiscriminate in its occurrence, affecting individuals across all ages, genders, and ethnicities [3].

The most crucial role in the pathogenesis of the disease is played by immunological factors. Under physiological conditions, an immunological balance is maintained within the hair follicle, and the guardians of this state include interleukin 10 (IL-10), transforming growth factor β-1 (TGF-β1), and alpha-melanocyte-stimulating hormone (α-MSH). Studies have shown that in alopecia areata, the expression of these substances is reduced, which leads to the hair follicle’s inability to defend itself against an immune system attack. Experimental studies have demonstrated that interferon-gamma (IFN-γ) and interleukins IL-2, IL-4, and IL-15 play a role in the autoimmune response in AA [1,2,3,4,5,6].

According to a 2020 expert consensus, increased risk factors for AA include a family history of the condition, and a personal history of other autoimmune disorders such as autoimmune thyroiditis or vitiligo [7,8,9]. Although these findings are supported by the literature, there is a lack of epidemiological and demographic data specific to Poland.

Several studies have emphasized the significant association between alopecia areata and mental health conditions, suggesting that stress and psychological factors play a crucial role in the development and progression of the disease [10]. However, further research is needed to better understand the relationship between immune reactions, stress, and the physiological characteristics observed in AA patients.

In December 2019, an outbreak of a novel coronavirus disease (COVID-19), caused by severe acute respiratory syndrome coronavirus 2 (SARS-CoV-2), was reported in Wuhan, China, quickly becoming one of the most significant global health challenges of the century [11].

The SARS-CoV-2 infection activates both cellular and humoral immune responses, influencing factors such as the regulation of CD8+/CD4+ T cell ratios, MHC class I and II molecules, and the production of IgM, IgA, and IgG antibodies [12]. Consequently, individuals with pre-existing comorbidities, particularly those with inflammatory and autoimmune disorders where the immune system is disturbed, are classified as at-risk groups. Moreover, since the pandemic’s onset, there has been a marked increase in the incidence of these conditions globally [13]. The literature emphasizes the crucial role of the COVID-19 pandemic as an immunological and stress trigger for the development of inflammatory and autoimmune diseases, for example type 1 diabetes [14,15]. Furthermore, numerous cases of adverse reactions to COVID-19 vaccines have been reported, among which alopecia areata is an uncommon, yet notable adverse event, as highlighted in several publications [16].

Given the impact of immune system dysregulation in alopecia areata, the emergence of COVID-19 and its effects on immune responses further highlights the need to explore the potential relationship between these two conditions. Hence, increasing attention is being directed toward the impact of the COVID-19 pandemic on the epidemiology of alopecia areata [14,15].

Although comprehensive national statistics on AA epidemiology in Poland are not yet available, data from the Polish National Health Fund (NFZ) indicate an upward trend in hospitalizations for benign dermatological conditions since the outbreak of the pandemic. Therefore, a detailed analysis of the epidemiology of alopecia areata in Poland is required.

The purpose of our study was to conduct a retrospective analysis of pediatric and adult patients diagnosed with alopecia areata, hospitalized in the dermatology department between 2015 and 2023. Our goal was to assess the influence of the COVID-19 pandemic on hospitalization rates for individuals affected by AA.

## 2. Materials and Methods

The following study was a retrospective cross-sectional analysis of all patients with a confirmed diagnosis of AA admitted to the Department of Dermatology, Paediatric Dermatology and Oncology Medical University of Lodz, Poland between 2015 and 2023. Patients were identified using the International Statistical Classification of Diseases 10th Revision (ICD-10).

The clinic database was filtered for electronic medical records fulfilling the above criteria. All charts were reviewed for the following variables: age, gender, residential location, number of hospitalizations and hospital stay duration. The extracted data were converted into digitized spreadsheets using Microsoft Excel 2007, Version 2604, compilation 19929.20136. The five-year period before the pandemic onset (2015–2019) was compared with the subsequent four years (2020–2023) to assess any changes in the selected factors.

Statistical analysis was performed using IBM SPSS Statistics for Windows, Version 29.0. IBM Corp. Released 2022. Armonk, NY: IBM Corp. Descriptive statistics were expressed as frequencies and percentages for categorical variables (age, gender, residential location, ICD-10 disease subtype). Mean ± standard deviation was used for numerical variables (hospitalizations number and duration). Statistical significance was assessed using an independent two-sample *t*-test, with a *p*-value threshold of < 0.05.

## 3. Results

Between 2015 and 2023, a total of 428 patients diagnosed with alopecia areata (AA) were enrolled in the study, comprising 256 children (59.81%) and 172 adults (40.19%). Among these patients, 292 (68.22%) were female and 136 (31.78%) were male. In the pediatric cohort, 163 (63.67%) were girls and 93 (36.33%) were boys. There were no statistically significant differences in gender distribution throughout the study period (χ^2^ = 3.83, df = 3, *p* = 0.28), with a median age of 39.13 years for adults and 8.66 years for children, as presented in Table 1.

Notably, in the four years following the COVID-19 pandemic outbreak in 2019, pediatric cases increased by 30.63%, rising from 111 to 145 patients, which was statistically significant (*p* = 0.034). Conversely, adult cases declined significantly, with 42 fewer adult patients reported in 2020 compared to 2019 (*p* = 7.36 × 10^−14^).

**Figure 1:**
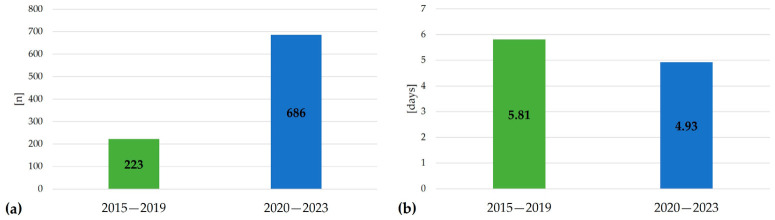
Comparison of hospitalization metrics for AA between 2015–2019 and 2020–2023: (**a**) Total number of hospital admissions and (**b**) mean duration of hospitalization in days.

**Figure 2:**
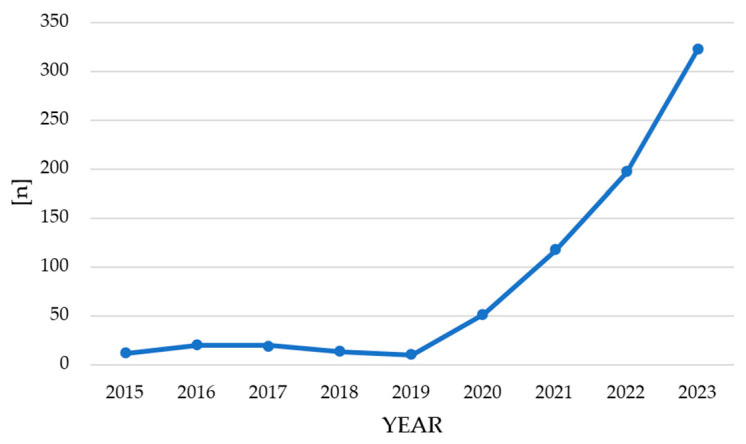
Annual number of hospital admissions for AA from 2015 to 2023.

Among patients hospitalized between 2015 and 2023, 178 (41.59%) had been hospitalized due to AA in the past, while 250 patients (58.41%) were diagnosed/hospitalized for the first time. The data indicate a marked increase in hospitalizations from 2020 to 2023, rising from 223 to 686 admissions (z-statistic = −21.72; *p* < 0.001) (Figure 1a). Of the 205 patients admitted in the post-pandemic period, 120 (58.54%) were diagnosed or hospitalized for AA for the first time, whereas the remaining 85 (41.46%) had been previously hospitalized for this diagnosis. The mean duration of hospitalization due to alopecia areata decreased by 15.15%, from 5.81 days (2015–2019) to 4.93 days (2020–2023), as presented in Figure 1b. Annual hospitalizations increased from 11 in 2015 to 323 in 2023 (*p* < 0.001), as shown in Figure 2.

**Figure 3:**
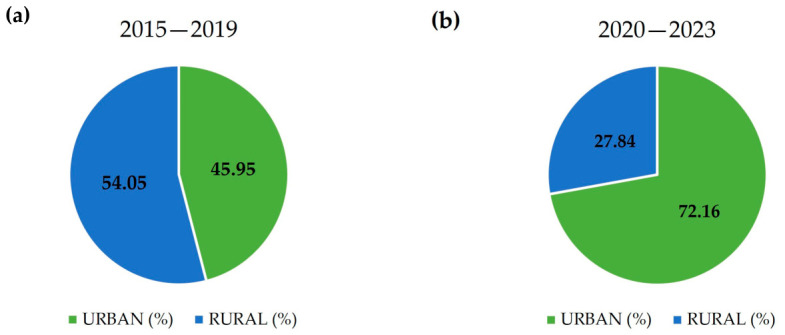
Geographical distribution of pediatric patients with alopecia areata: (**a**) Percentage of patients residing in urban vs. rural areas during 2015–2019; and (**b**) percentage of patients residing in urban vs. rural areas during 2020–2023.

Geographically, between 2015 and 2019, 45.95% of pediatric patients resided in urban areas, while 54.05% lived in rural settings, as shown in Figure 3a. This distribution changed significantly from 2020 to 2023, with 72.16% of children residing in urban areas and 27.84% in rural areas (z = −4.31, *p* < 0.001), as illustrated in Figure 3b.

A detailed analysis of disease classification according to the International Statistical Classification of Diseases, 10th Revision (ICD-10) revealed a significant rise in cases of alopecia areata totalis, both in the overall cohort (t = −10.08, *p* = 2.03 × 10^−5^) and among pediatric patients (t = −9.46, *p* = 3.09 × 10^−5^).

The most common comorbidities were atopic dermatitis [*n* = 35; (8.18%)], autoimmune thyroiditis [*n* = 27; (6.34%)], and diabetes [*n* = 33; (7.71%)].

## 4. Discussion

Since the beginning of the COVID-19 pandemic, there have emerged numerous reports of both new-onset AA and its recurrence or exacerbation following SARS-CoV-2 infection [14,17,18,19,20]. There exist several immunological hypotheses which may explain the correlation between the two conditions; nevertheless, the exact mechanism behind the phenomenon remains unclear due to the limited amount of data. In addition, the multifactorial aetiology of AA itself makes it difficult to distinguish SARS-CoV-2 infection and pandemic-related psychological distress from other causative factors of the disease [17].

As far as the patients’ epidemiological profile is concerned, in the present sample women and girls were affected by AA more frequently than men and boys, constituting almost 70% of the patients. These results appear to be in line with data from the Global Burden of Disease Study 2019, which likewise shows slight female predominance [21]. This tendency is also visible in the reports of AA associated specifically with COVID-19 [14,17]. The gender bias, however, may be attributed to a higher female concern regarding hair loss, due to which they are more willing to seek help and therefore participate in clinic-based studies [22,23,24].

In the adult cohort, the median age was 39.13 years, which is comparable with the data from other studies done in Israel and in the US [25,26]. Similarly to Harries et al., we noted that adult females’ mean age was relatively higher (46) when compared to adult males (31) [27]. Children median age in our study equaled to 8.66 years, which corresponds to the value observed by McKenzie et al. (8.6 years). The authors also report that girls were affected by AA more frequently than boys, which was also observed in our pediatric cohort [28]. Of note, our analysis revealed a 13.81% mean age decrease among adult males (from 36.07 to 31.06 years) after the COVID-19 pandemic onset. Since 2019, however, the amount of epidemiological data regarding AA is extremely limited. To the best of our knowledge, none of the authors has elaborated on such trend in the literature available to date.

After the pandemic onset, the number of AA-related hospitalizations in our department surged by 207.62%, rising from 223 to 686 admissions. Importantly, in our cohort, the post-pandemic period was characterized by a predominance of newly diagnosed patients, with the majority of hospitalizations after 2020 representing first-time admissions for AA. This observation may reflect the impact of the pandemic period on the emergence of new-onset alopecia areata cases. According to the Polish National Health Fund (NFZ) data, a two-fold increase in hospital stays associated with AA was observed in the entire country since 2019 [29]. The concordance between our single-center data, regional statistics, and nationwide reports from the National Health Fund (NFZ) suggests that the increase in alopecia areata hospitalizations observed in our department reflects a broader epidemiological trend rather than a local anomaly. Nationwide AA hospitalizations rose from 232 cases in 2017 (1.01% of all dermatological hospitalizations) to 418 in 2019 (1.77%), declined temporarily in 2020 (258 cases), and subsequently increased to 792 in 2023 (3.08%), representing more than a threefold rise compared to 2017 [29]. A similar pattern was noted regionally, where total dermatological hospitalizations decreased from 1725 in 2019 to 817 in 2020, paralleling the nationwide decline from 21,094 to 11,910 cases, followed by post-pandemic recovery. Although inclusion of additional centers would enhance epidemiological precision, pandemic-related restrictions, increased clinical workload, and data harmonization challenges limited multicenter analysis. Nevertheless, the incorporation of representative NFZ data partially mitigates this limitation and supports the generalizability of our findings. Our observations underscore the growing significance of alopecia areata within the broader context of dermatological health care.

As for the mean duration of hospitalization, we observed a decrease of 15.15% (from 5.81 to 4.93 days). These findings are congruent with the ones published by Brzychcy et al. [22] as well as with the above-mentioned national data [29].

Although unspecified AA remained the most frequently diagnosed disease subtype in both time periods analyzed in our study, after the onset of the COVID-19 pandemic we observed an increase in the diagnosis of severe disease subtypes (AT and AU), from a combined 11.66% between 2015 and 2019 to 20.00% between 2020 and 2023. Similarly, Kim et al. noted higher diagnosis rates of diverse disease subtypes (including AT and AU) in COVID-19 affected AA patients when compared to an uninfected control group [18].

According to the literature, AA incidence is higher in people from areas of higher social deprivation [27]. Similarly, between 2015 and 2019, pediatric patients from rural areas were hospitalized in our department more frequently than urban residents and constituted 54.05% of the patients. Since the pandemic onset; however, the trend reversed, as between 2020 and 2023 children from urban areas constituted the majority of AA-affected patients (72.16%). The observed post-pandemic shift may reflect several interacting factors. First, differences in healthcare accessibility between urban and rural areas during the COVID-19 pandemic could have influenced referral patterns, as specialized dermatological services are more readily available in urban centers. Second, pandemic-related psychosocial stressors, including prolonged school closures, social isolation, and increased screen time, may have been more pronounced in densely populated urban environments and could have contributed to disease onset or exacerbation. Easier contraction of the SARS-CoV-2 virus due to more difficult social distancing in densely populated areas may also have contributed to the observed change. It is also noteworthy that urban living itself generally constitutes an independent factor promoting AA incidence [27]. Environmental factors such as higher levels of air pollution in urban areas have been hypothesized to influence autoimmune and inflammatory conditions, however, direct evidence for alopecia areata remains limited. Of note, the shift toward a higher proportion of urban patients was not observed in the adult cohort of our study. Likewise, in a study published by Kim et al. there was no significant difference between the residential location of COVID-19-positive AA-affected patients and the uninfected control group [18]. Further multicenter and population-based investigations are warranted to clarify whether the observed shift represents a true epidemiological change or reflects healthcare access dynamics.

Our analysis presents several limitations which merit attention. Due to the retrospective character of the cohort, we could not capture patients affected by AA who did not seek medical care. For this reason, the results may overrepresent cases of severe AA, as these patients are more likely to seek help. Moreover, the study design did not involve COVID-19 testing of the patients, nor did it include a COVID-19-negative control group. Vaccination status was not routinely recorded as part of the anamnesis during dermatological consultations and therefore could not be tracked retrospectively. Psychological stress levels were not objectively measured via questionnaires or cortisol levels; thus, it is not possible to document a possible increase after the pandemic onset. Given that hair loss may act as an independent stressor capable of exacerbating AA, it is also impossible to objectively distinguish whether a patient’s stress was caused by the pandemic or the disease itself. The study therefore demonstrates a possible correlation, yet cannot establish causality between AA and the COVID-19 pandemic.

## 5. Conclusions

In conclusion, our research reveals a profound increase in hospitalizations and a diversification of disease subtypes among patients with alopecia areata (AA) in the aftermath of the COVID-19 pandemic. These findings not only underscore the immediate impact of the pandemic on dermatological health but also align with a growing body of evidence suggesting a potential correlation between SARS-CoV-2 infection and the development, recurrence, or exacerbation of AA. The significant rise in severe subtypes of AA, such as alopecia totalis and universalis, further emphasizes the need for a nuanced understanding of the mechanisms at play. This evolving landscape of AA in the context of the pandemic highlights the complexity of autoimmune responses and their potential triggers, including viral infections.

As we strive to improve dermatologic treatment outcomes, a deeper exploration of this association is essential. Future research endeavors must aim to elucidate the intricate relationship between COVID-19 and alopecia areata, potentially leading to more targeted and effective therapeutic strategies. By fostering a comprehensive understanding of these interconnected conditions, we can better equip healthcare providers and improve the quality of care for patients grappling with the multifaceted challenges of alopecia areata in a post-pandemic world.

## Figures and Tables

**Table 1:** Demographic characteristics of the study cohort.

Characteristic	Total Cohort (*n* = 428)	Pediatric Patients (*n* = 256)	Adult Patients (*n* = 172)
Age, years (median)	-	8.66	39.13
Female sex, *n* (%)	292 (68.22)	163 (63.67)	129 (75.00)
Male sex, *n* (%)	136 (31.78)	93 (36.33)	43 (25.00)

## Data Availability

The datasets generated and/or analyzed during the current study are not publicly available due to ethical reasons but are available from the corresponding author on reasonable request. All shared data will be provided in de-identified form to ensure participant privacy.

## Abbreviations

The following abbreviations are used in this manuscript:

AA	Alopecia Areata
SARS-CoV-2	Severe Acute Respiratory Syndrome Coronavirus 2
COVID-19	Coronavirus Disease 2019
ICD-10	International Statistical Classification of Diseases and Related Health Problems, 10th Revision
IL-10	Interleukin 10
TGF-β1	Transforming Growth Factor β-1
A-MSH	Alpha-Melanocyte-Stimulating Hormone
IFN-γ	Interferon Gamma
AT	Alopecia Totalis
AU	Alopecia Universalis
NFZ	Polish National Health Fund
